# A Case of Successful Operation for the Excision of the Ossified Cartilages and Anterior Extremities of Two Carious Ribs, and the Lower Portion of the Sternum

**Published:** 1831

**Authors:** Geo. McClellan

**Affiliations:** Professor of Surgery in Jefferson College


**Art. III.**—*A case of successful operation for the excision of the ossified cartilages and anterior extremities of two carious ribs and the lower portion of the sternum*, by Geo. McClellan, M. D., Professor of Surgery in Jefferson College.

*Philadelphia, Jan.* 27, 1831

To Daniel Drake, M. D.

My Dear Sir—Herewith I enclose the autograph letter of a patient, in the history of whose case you appear to have taken a deep interest. The bony specimens which were preserved by his family after the operation, have just come to hand, and I will send them to you before you leave the city on your return to Cincinnati.

As near as I can judge, from a recollection of his appearance at the time of the operation, I should suppose that the patient was about 60 years old. He was a very large person and corpulent; and his chest was at least as full and broad as I recollect ever to have observed in the case of any other individual. This will account for the unusual length of the cartilages of the ribs which were excised, and for the extent of the incisions which I was obliged to make in the superincumbent soft parts.

In addition to the symptoms enumerated in the enclosed letter, I must not omit to state that I was particularly struck with the appearance of great oppression and difficulty of respiration under which the patient had been laboring for a considerable period of time before I visited him. On passing a probe through a fistulous opening, situated a short distance from the right edge of the sternum, I was also surprised to find the two adjacent costal cartilages presenting hard and rough bony surfaces, along both of which I could convey the probe its whole length toward the right side. This circumstance immediately convinced me that the disease had originated from an ossification of these cartilages, and a consequent inflamation in or around them, which had finally produced a detachment of the perichondria, and a caries, if not a necrosis, of both substances.

I commenced the operation by carrying a deep incision from the edge of the sternum outwards to the distance of 7 or 8 inches along the intercostal space between the 5th and 6th ribs.

The edges of this incision were then dissected up, until the surfaces of both these ribs were fully exposed. They were evidently ossified cartilages presenting the appearance of worm-eaten caries throughout their whole length. The perichondrium was completely detached from the external surface of both, and thickened by induration of the surrounding cellular substance. I encountered but little difficulty, therefore, in detaching this envelope and pushing it backwards from the upper and lower edges of each rib, by the use of the handle and occasionally the blade of a common scalpel. The attenuated intercostal muscles and tendinous fibres, were of course detached along with these membranes. The bones were then left sufficiently bare to admit of the application of Hey's saw, which I repeated twice on each rib. With a common elevator I then pried up the bones from their outer toward their sternal attachments;—in accomplishing which, only two or three applications of the knife were required to cut away adhesions to the indurated mass of membranes and cellular tissue within. As the projecting extremities of the ribs did not appear perfectly sound, I extended the incision a little further outwards, and cut away about 1 1-2 inches more from their substance. The lower portion of the sternum presenting a similar appearance of disease, I also enlarged the incision inwards, and sawed away a considerable portion of that bone. On examining the exposed surface, there appeared a convoluted bony plate, surrounded by some indulated cellular tissue and medullary looking matter, pressing upon the pleura costalis, and intruding upon the cavity of the chest. I mistook this at first for the extremity of the seventh rib, which I supposed had been turned inwards and upwards behind the corresponding cartilages of the 5th and 6th ribs. On a closer inspection, however, I became convinced that it proceeded from ossification of a detached portion of the perichondrium of the 6th rib which had attempted to set up a sequestrating process around the cartilage. I succeeded very easily in prying up the whole of this mass with an elevator which detached it cleanly from the pleura, with the same kind of sound that is emitted from a birch log when it is stripped of its bark.

The intercostal fibres, both muscular and tendinous, were chiefly removed along with the bony plate, and the pleura was then left as thin as natural, rising and falling alternately with the efforts of respiration. As the hemorrhage from the torn branches of the intercostal and mammary arteries ceased almost instantaneously, I soon closed up the incision in the integuments, and retained them in apposition by interrupted sutures and adhesive plasters. A broad compress and bandage completed the dressing, and I left the patient directly afterwards in the care of my friend, Dr. Gloninger. The final result, together with a satisfactory detail of all the leading circumstances you have in the enclosed letter. Truly yours,

GEO. McCLELLAN.

---

*Lebanon, Penn., Nov.* 22, 1830.

Dear Doctor—

Having understood that you expressed a desire to be furnished with a detail of my case, prior and subsequent to the operation you performed, I will give it with the greatest pleasure: at the same time, I cannot, in justice to my feelings, but reiterate my gratitude to you for the benefits you have conferred on me, by your skill, in restoring me to my wonted health.

In the fall of 1826, I experienced more or less pain in my right side, extending along the fifth and sixth ribs to the sternum, accompanied with a tumefaction of the integuments. This continued for several months, without any abatement, until a fistulous opening appeared between the fifth and sixth ribs, about two inches from the breast bone, discharging, daily, a quantity of pus. My health, from this period, began rapidly to decline; the pain augmenting, my strength failing, and my emaciation becoming perceptible to myself and friends. My debility was now so great, as to cause my constant confinement to bed. The Syrup of Cuisinier was now recommended to me, and, after taking about ten bottles of it, without experiencing any benefit whatever, from its use, I placed myself under the care of a German practitioner, Dr. Dostor, who assured me he could restore me to my accustomed health.

He purged me daily for nearly four weeks with some purgative pills, and applied discutient plasters to the diseased part. Under this mode of treatment, finding no amelioration of my sufferings, but rather an aggravation of them, I consulted Dr. John W. Gloninger, of this place, who, upon an examination of the affected part, with a probe, pronounced my disease to be a caries of the 6th and 5th ribs, together with the lower portion of the breast bone, and advised an operation, to which I did not consent until my hectic symptoms became alarming, and my debility so great, that I despaired of being restored to my health by other remedial means.

In this situation you found me the latter part of October, 1827, when you performed the operation. Soon after the operation was performed, the hectic fever left me, and my strength slowly returned, though I could not leave my room for nearly twelve months thereafter. About a fortnight after the operation, Dr. Gloninger gave me three grains of the blue pill, united with a half grain of ipecacuanha, every night at bed time, followed by a slight laxative every other morning, and accompanied by a liberal use of the decoction of sarsaparilla.

With this course he persevered, until I became slightly salivated, and established, at the same time, a caustic issue on the centre of the sternum, opposite the inner edge of the incision.

He did not allow the issue to heal for about four or five months, at the expiration of which time the fistulous opening closed, which had appeared near the lower extremity of the sternum, soon after the incision had cicatrized. I now commenced with a decoction of the bark of the prunus virginiana, and continued with its use for about ten months with occasional intermissions, until my health was perfectly re-establised. It is now as good as it ever was, and I find that I am able to attend to the duties of register and clerk of the orphan's court of Lebanon County, without the least pain or inconvenience arising from the removal of a part of two of my ribs, and a portion of my breast bone. Very respectfully,

JNO. UHLER.

